# The differentially expressed proteins related to clinical viral encephalitis revealed by proteomics

**DOI:** 10.1002/ibra.12036

**Published:** 2022-05-04

**Authors:** Qian Wang, Shan‐Shan Yan, Jun‐Yan Zhang, Ruo‐Lan Du, Lu‐Lu Xue, Juan Li, Chang‐Yin Yu

**Affiliations:** ^1^ Affiliated Hospital of Zunyi Medical University Zunyi Guizhou China; ^2^ Southwest Medical University Luzhou Sichuan China; ^3^ Institute of Neurological Disease and Department of Anesthesiology, Translational Neuroscience Center, West China Hospital Sichuan University Chengdu China; ^4^ Kunming Medical University Kunming Yunnan China

**Keywords:** differentially expressed proteins, proteomics, viral encephalitis

## Abstract

To screen out the prospective biomarkers of viral encephalitis (VE), analyze the biological process and signaling pathways involved by differentially expressed proteins (DEPs). A total of 11 cerebrospinal fluid (CSF) samples with VE and 5 with non‐nervous system infection were used to perform label‐free proteomic techniques. Then, the bioinformatic analysis of DEPs was applied by Interproscan software. Moreover, 73 CSF samples in the VE group and 53 in the control group were used to verify the changes of some DEPs by enzyme‐linked immunosorbent assay (ELISA). Thirty‐nine DEPs were identified, including 18 upregulated DEPs and 21 downregulated DEPs. DEPs were mainly enriched in cell adhesion molecules by Kyoto Encyclopedia of Genes and Genomes analysis pathway analysis. The DEPs related to axon tissue were obviously downregulated and the most significant downregulated proteins were neurexin 3, neurofascin, and neuroligin 2 (NLGN2). Moreover, the protein expression of NLGN2 in the VE group was significantly higher than that in the control group by ELISA. The correlation analysis of NLGN2 in the VE group revealed that there was a weak positive correlation with CSF protein and a weak negative correlation with CSF chloride. The clinical VE may be closely related to NLGN2 and the cell adhesion molecule pathway.

## Graphical abstract

To screen out the prospective biomarkers of viral encephalitis (VE), unlabeled proteomic tests and gene network analysis were performed on 11 VE cerebrospinal fluid (CSF) samples and 5 CSF samples from non‐nervous system infections. Enzyme‐linked immunosorbent assay  was used to detect differentially expressed proteins, and then the sample volume was increased to 73 CSF samples in the VE group and 53 CSF samples in the control group to verify neuroligin 2 (NLGN2) protein. NLGN2 remarkably expressed, compared with the control group, in the CSF with VE. (B) Comparison of *NFASC* protein between VE and control groups (20 samples in each group). (C) Comparison of *NLGN2* protein between VE and control groups (with an increase in sample volume, 73 cases in the VE group, and 53 cases in the control group). ELISA, enzyme‐linked immunosorbent assay; *NFASC*, neurofascin; *NLGN2*, neuroligin 2; *NRXN3*, neurexin 3; VE, viral encephalitis.

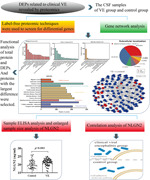

## 1 INTRODUCTION

Viral encephalitis (VE) is an infectious disease of the central nervous system (CNS) caused by multiple viral infections, which is on account of the viral retrograde invasion of CNS brain parenchyma and meninges through nerve endings or blood.^1^, ^2^, ^3^ The worldwide incidence of VE ranges from 3.5/10,000 to 7.4/10,000. It is common in the young and the elderly, with a rising incidence.^4^ Studies have reported that there are about 100 kinds of viruses causing VE, among which enterovirus, herpes simplex 1 (HSV‐1), herpes simplex 2 (HSV‐2), and varicella‐zoster virus are the most common types that cause encephalitis in children and adults.^5^, ^6^, ^7^ The body produces an inflammatory reaction to damage the nervous function, which manifested as fever, headache, epileptic seizure, mental and behavioral abnormalities, focal nervous function defect, and even consciousness disorder in serious cases after the virus infects the nervous system. The current diagnosis mainly depends on clinical manifestations, imaging, cerebrospinal fluid (CSF) cytology, etiology, and serological examination. Etiological examination is one of the important diagnostic methods of this disease, mainly including viral antibody detection, CSF virus culture, and polymerase chain reaction (PCR). Nevertheless, due to its low sensitivity and specificity, there are still chances of missed diagnosis and misdiagnosis.^8^, ^9^ Over the past 20 years, molecular etiological tests, as a complement to serological tests and immunohistology, have improved the diagnosis of CNS. However, these methods are limited by their clinical technical pertinence. In addition to clinical technical limitations, it is also affected by the type of samples, such as CSF, brain biopsy, the time of sample collection, the pathogenic mechanism of the virus, and so on. Despite there are advances in technology, the etiology of approximately 50% of encephalitis, meningoencephalitis, and meningitis remains unknown.^10^, ^11^, ^12^, ^13^ Meanwhile, the atypical early presentation and infection can easily lead to different degrees of nervous system sequelae, with a high incidence and mortality. So, an early diagnosis and effective drug treatment are of great significance.

Many studies have reported that some molecules in CSF can be used as potential biomarkers for some CNS and biological targets in drug therapy. For example, PR‐39 is significantly increased in CSF of streptococcal meningitis piglets, which degrades neutrophilic DNA by inhibiting bacterial nuclease.^14^ Alzheimer's disease (AD) decreased insulin concentration in CSF may be involved in the pathogenesis of AD.^15^ The expression of CSF central nervous‐specific protein (S100β), glial fibrillary acidic protein, neuron‐specific enolase, and myelin basic protein in VE is higher than that in normal subjects, which can be monitored clinically to evaluate the severity degree.^16^ These study results suggest that the discovery of differentially expressed proteins (DEPs) in CSF may provide an important candidate biological target for the diagnosis, pathogenesis, and drug therapy of CNS.

As a method of mass spectrometry, label‐free proteomics techniques compare the times of mass spectrometry analysis or the peak strength of polypeptides after enzymatic hydrolysis of proteins in samples for quantitative analysis. Unlike other methods for protein quantification, it does not use stable isotope chemical binding to label proteins. It is widely used to determine the relative number of proteins in two or more biological samples. In recent years, it has been widely used to find out some “disease‐specific protein molecules” and reveals the pathogenesis of diseases, which provides more research ideas and a theoretical basis for studying the mechanism and treatment of diseases.^17^, ^18^ To date, CSF proteomics has rapidly developed into a new technique for diagnosing and investigating the mechanisms of diseases, especially neurological‐related diseases, such as Alzheimer's disease, multiple sclerosis, Parkinson's disease, chronic nerve headache, acute brain injury, and mental disorders.^19^, ^20^, ^21^, ^22^ At present, there are great limitations in the diagnosis of VE/viral meningitis. It lacks strong positive evidence, having many missed and misdiagnosis cases. There is little research report on the analysis of CSF protein (CSF Pro) of VE. The disease has a high incidence in China. Therefore, the label‐free proteomics technology was applied to analyze the CSF of patients with VE at protein so as to find new potential biomarkers for VE. Hence, a new mechanism could potentially be elucidated, which is of great significance to provide the diagnostic basis for a deeper understanding of VE and the promotive accuracy of diagnosis.

## 2 MATERIALS AND METHODS

### 2.1 Main experimental materials

#### 2.1.1 Study time and subjects

The research period was from November 2020 to February 2021. This study was approved by the Ethics Committee of the Affiliated Hospital of Zunyi Medical University and the Third Affiliated Hospital of Zunyi Medical University (First People's Hospital of Zunyi City). The informed consent was signed by patients or their families.

The cases were hospitalized patients with VE and patients with non‐nervous system infection clinically (control group), diagnosed in the Department of Neurology of the Affiliated Hospital of Zunyi Medical University and the Third Affiliated Hospital of Zunyi Medical University.

#### 2.1.2 Sample size calculation

According to the preliminary experimental results (20 samples in each group), the sample size formula *n* = 2 × [(*α* + *β*)*σ*/*δ*]^2^ was used to calculate the sample size. Power = 0.8 and *α* = 0.05 were selected (both sides), and the sample size was estimated to be about 60 cases in each group.

#### 2.1.3 Experimental technical route

**Figure 1:**
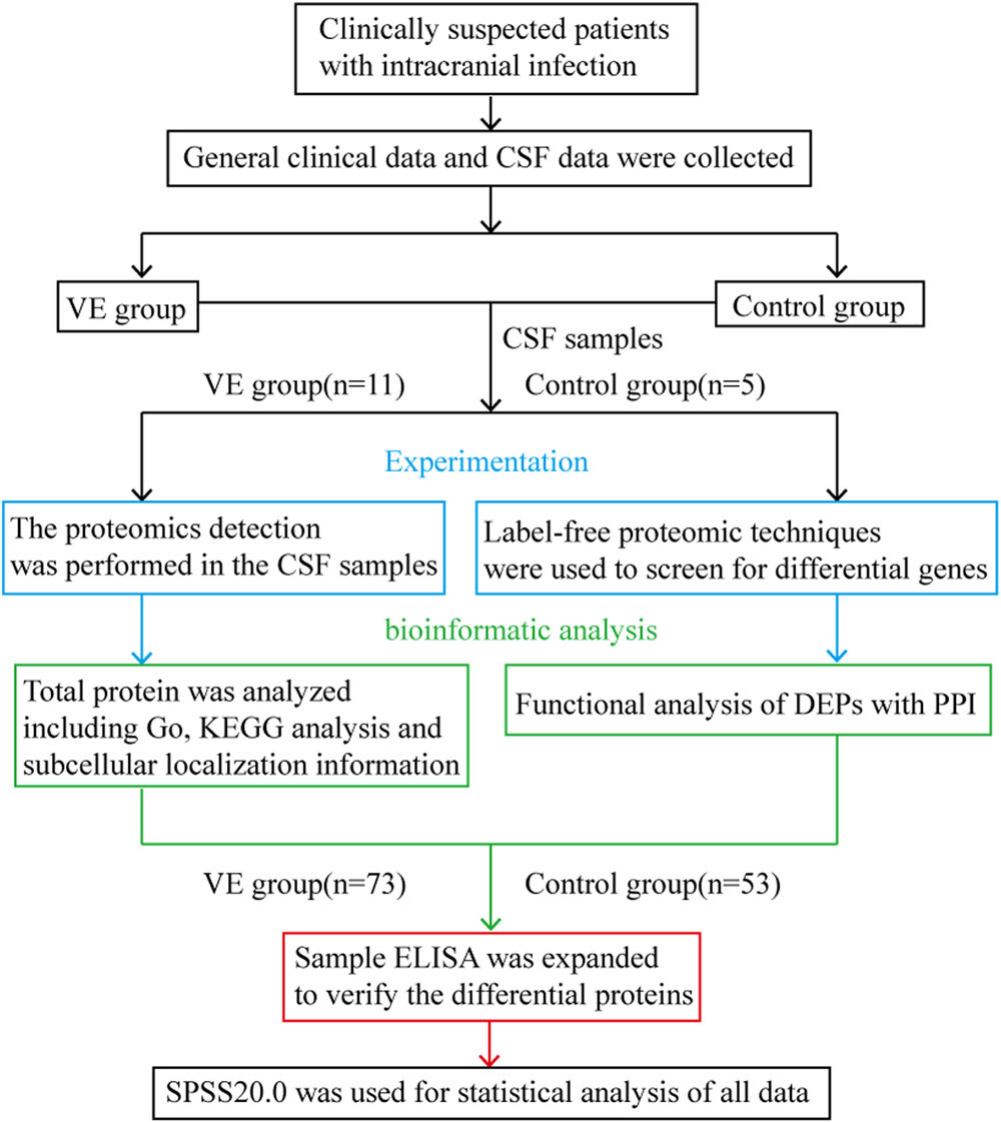
Experimental route. CSF, cerebrospinal fluid; DEP, differentially expressed proteins; ELISA, enzyme‐linked immunosorbent assay; GO, gene ontology; KEGG, Kyoto Encyclopedia of Genes and Genomes analysis; PPI, psychophysiological interaction; VE, viral encephalitis. [Color figure can be viewed at wileyonlinelibrary.com]

The CSF of 11 patients with VE and 5 with non‐nervous system infection were collected, and the differential changes of proteins were analyzed by label‐free proteomics technique to obtain the significant DEPs. Furthermore, we used an enzyme‐linked immunosorbent assay (ELISA) to verify the identified proteins (Figure 1).

#### 2.1.4 Inclusion criteria and exclusion criteria

The inclusion criteria and exclusion criteria are shown in Table 1.

**Table 1:** Inclusion criteria and exclusion criteria

	Entry criteria	Exclusion criteria
VE group	All of them met the diagnostic criteria for CNS in Clinical Neurology edited by Huang Ruxun: (1) Patients have acute or subacute onset and suspected cold infection symptoms before onsets, such as fever, general malaise, pharyngalgia, and so on. (2) Focal or diffuse neurological symptoms, such as epilepsy, consciousness disorder, mental and behavioral abnormalities, hemiplegia, aphasia, cranial nerve paralysis, ataxia, and so on. (3) Meningeal irritation sign is positive or negative. (4) Signs of infection at primary sites other than the central nervous system, such as measles and chickenpox, may appear. (5) CSF of major patients showed that blood white blood cells are normal or increased, CSF pressure is normal or increased, white blood cells are increased (mainly lymphocytes), sugar and chloride are normal or slightly higher, protein is slightly increased. CSF of a few patients can be completely normal. (6) EEG is normal and shows a focal abnormality, or diffuse slow wave and other changes. (7) Craniocerebral imaging can show inflammatory changes and/or meningeal enhancement, or normal. (8) Antiviral therapy is effective.	The subjects in each group had no other infectious diseases, no immune system diseases, no serious liver and kidney diseases, no recent history of blood transfusion, no history of immunosuppressive agents and corticoids, and no history of tumor.
Control group	CSF of patients diagnosed with migraine, cerebral arterial insufficiency, hysteria, secondary headache, and so on is the control group. (1) There is a suspicious history of catching a cold. (2) Clinical manifestations include dizziness and headache, which shows suspected central nervous system infection. (3) There were no slow wave changes in EEG, and no abnormalities were found in CSF pressure, biochemical, and routine examination results. (4) There was no positive sign in the nervous system examination. (5) Cranial CT/MRI excludes cerebrovascular diseases, intracranial space‐occupying lesions, and other manifestations. (6) Without antiviral treatment, patients only improve by symptoms of symptomatic treatment.	The subjects in each group had no other infectious diseases, no immune system diseases, no serious liver and kidney diseases, no recent history of blood transfusion, no history of immunosuppressive agents and corticoids, and no history of tumor.

Abbreviations: CT/MRI, computed tomography/magnetic resonance imaging; CSF, cerebrospinal fluid; DEP, differentially expressed proteins; EEG, electroencephalogram; ELISA, enzyme‐linked immunosorbent assay; GO, gene ontology; PPI, psychophysiological interaction; VE, viral encephalitis.

### 2.2 General clinical information collection

General clinical data of patients were collected, including name, sex, age, electroencephalogram, cranial computed tomography (CT)/magnetic resonance imaging, chest X‐ray, chest CT, blood white blood cells (WBC), blood neutrophils absolute value (NEUT), blood lymphocyte absolute value (LY), CSF pressure, the CSF leukocyte (CSF WBC), CSF chloride (CSF Cl), CSF Pro, CSF glucose (CSF Glu), hospitalization days, and so on.

#### 2.2.1 Collection of CSF specimens and total protein extraction

Two millimeters of CSF from patients suspected of intracranial infection were collected by lumbar puncture, and stored in the −80 refrigerator (China Acoma Company). The CSF samples were lyophilized by a freeze dryer (Labogene), ground into powder in a liquid nitrogen environment, and transferred into a centrifuge tube to precool by liquid nitrogen. The amount of protein lysate that was prepared appropriately with 100 mM ammonium bicarbonate (Sigma Corporation), 8 M urea (China National Pharmaceutical Group), and 0.2% sodium dodecyl sulfate (China Medical Group), pH = 8 was added and mixed. It was cracked for 5 min in an ultrasonic cell crusher (China Ningbo Xinzhi Biotechnology Co., Ltd.) with an ice water bath and then centrifuged (4°C, 12,000*g*, 15 min). The supernatant was taken by a pipettor (Eppendorf) and 1% dithiothreitol was added (Sigma Company) by volume ratio and thoroughly mixed by Vortex Mixer (China Photosynthetic Biotechnology Co., Ltd.). The liquid was bathed at 56°C for 1 h. Iodoacetamide (Sigma Corporation) was added by volume ratio and reacted at room temperature for 1 h without light. Four times the volume of precooled acetone was added and precipitated at −20°C for at least 2 h. It was centrifuged for 15 min (4, USA, 12,000*g*) and the precipitate was collected. After that 1 ml of precooled acetone was added to the precipitate, resuspended, and cleaned. Then, it was centrifuged for 15 min (4°C, 12,000*g*) by the low‐temperature, high‐speed centrifuge (Eppendorf). The precipitate was collected and then air‐dried. An amount of proteolytic solution (6 M, 100 mM TEAB, pH = 8.5) was appropriately added to dissolve the protein precipitate.

#### 2.2.2 Protein quantification

Bradford Protein Quantitative Kit (Shanghai Biyuntian Biotechnology Co., Ltd.) was used to prepare bovine serum albumin (BSA) standard protein solution according to the instructions, and the gradient range was 0–0.5 g/μl. The BSA standard protein solution and the diluted sample were added to the 96‐well plates. Three duplicate wells were made. G250 staining solution of 180 μl was added quickly and placed at room temperature for 5 min. The absorbance at 595 nm was measured. The standard curve was drawn and the protein concentration of the sample was calculated. Twenty micrograms of protein samples were taken for 12% sodium dodecyl‐sulfate polyacrylamide gel electrophoresis by Electrophoresis Instrument (Bio‐Rad Company) and Electrophoresis Tank (Bio‐Rad Company). The concentration of gel electrophoresis was at 80 V and 20 min, and the separation of gel electrophoresis was at 120 V and 90 min. Coomassie brilliant blue R‐250 staining was performed and decolorized until the bands were clear after electrophoresis.

### 2.3 Proteolysis

One hundred and twenty micrograms of protein samples were added with trypsin for digestion, and then the volume was replenished to 100 μl by 100 mM TEAB buffer (Sigma Corporation), and enzymatic digestion at 37°C. Trypsin (Promega) and CaCl_2_ were added proportionally for a night. Then, they were adjusted to a pH < 3, mixed well, and centrifuged with 12,000*g* for 5 min at room temperature. The supernatant was slowly desalted through the C18 column and then washed with a cleaning solution, which was prepared with 0.1% formic acid (Thermo Fisher, Inc) and 3% acetonitrile (Thermo Fisher, Inc) three times. After that, moderate eluent (0.1% formic acid, 70% acetonitrile) was added for washing. The filtrate was collected, lyophilized, and stored at a low temperature (−20°C) for later use.

#### 2.3.1 Protein mass spectrometry

The test was accomplished by Beijing Nohe Zhiyuan Technology Co., Ltd. First, the lyophilized proteases extracted from CSF were resuspended in Buffer A, which was prepared with 100% LC‐MS Ultra‐Pure Water (Thermo Fisher, Inc) and 0.1% formic acid. The EASY‐nLCTM1200 nm Upgrade UHPLC system (Thermo Fisher, Inc) was connected to the Q ExactiveTM HF‐X mass spectrometer (Thermo Fisher, Inc). The parameter precolumn was adjusted to a self‐made precolumn (2 cm × 75 μm, 3 μm), and the analytical column was self‐made (15 cm × 150 μm, 1.9 μm). The flow column was washed with Buffer A for 3 min, and the elution conditions were increased by using Buffer B (80% acetonitrile, 0.1% formic acid) to 90% by a gradient of 4% within 60 min. Data were collected by data‐dependent acquisition mode, with a scanning range of mass spectrum selected as 375–1800*m*/*z* (primary resolution: 60,000*; m*/*z*: 200; secondary resolution: 15,000; *m*/*z*: 200).^23^ To prevent the repeated selection of peptides, the dynamic exclusion range was set as 20 s. The original data were collected by Q‐Exactive, and all the result spectra were searched by Proteome Discoverer 2.2, which was based on the human protein database. The immobilized modification was alkylation modification of cysteine, the variable modification was methionine oxidation modification, and the N‐terminal was acetylation modification, allowing a maximum of 2 deletion points. To improve the quality of the analysis results, PD2.2 software further filtered the retrieval results. Peptides with a confidence interval greater than 99% were trusted peptide spectrum matches (PSMs). Only credible peptides and proteins were retained and false discovery rate (FDR) verification was performed. Peptides and proteins whose FDR was greater than 1% were removed. *T* test was used for protein quantitative statistical analysis results. The proteins with significant quantitative differences (*p* < 0.05, | log_2 _fold‐change| > 1.2) between the experimental group and the control group were defined as DEPs. We defined proteins that are significantly expressed or only present in VE CSF as VE‐DEPs.

### 2.4 Data analysis

Gene Ontology (GO) enrichment analysis of total protein was performed using Interproscan software. The corresponding biological process (BP), molecular function (MF), and cellular component (CC) of the retrieved proteins were analyzed.^24^ Besides, Kyoto Encyclopedia of Genes and Genomes analysis (KEGG) analysis and subcellular localization analysis were performed for the total protein, and cluster analysis was performed for the relative protein content in the samples. Protein comparison of CSF Pros in the VE group and the control group was performed by the intensity‐based absolute‐protein‐quantification (IBAQ) method.^25^ In simple terms, it is the strength value of each protein divided by the number of theoretical peptides that produce protein during trypsin digestion. The differential multiple for each protein is calculated by taking the logarithm of the average IBAQ value against the corresponding IBAQ value in the control group. To calculate the statistical significance, 0 IBAQ value was replaced with 1, and the IBAQ values of each protein were analyzed by *T* test. The DEPs were performed by volcano map analysis, subcellular localization analysis, BP, InterPro protein domain (IPR), and KEGG pathway enrichment analysis. The potential protein–protein interactions were predicted by the STRING and DB software.^26^, ^27^

### 2.5 Detection of neurexin 3 (*NRXN3*), neurofascin (*NFASC*), and neuroligin 2 (*NLGN2*) proteins by ELISA

CSF sample was taken out of the refrigerator at −80°C, centrifuged 200 μl in a precooled 4°C centrifuge for 20 min (2000*g*), and the supernatant was removed and labeled for later use. The ELISA kit was removed from the −20°C refrigerator and placed at room temperature for 60 min to dissolve the reagent. The number of plates required for testing was determined. Three auxiliary holes were made for each sample with a blank well, standard well, and sample well set up, respectively. The diluted standard samples (16, 8, 4, 2, 1, and 0 ng/ml) were added to six standard wells, with 50 μl to each. One well was only added with sample diluent as a blank control. The remaining wells were added with 40 µl sample diluent and 10 µl CSF sample, and mixed gently. One hundred microliters of enzyme labeling reagent (*NRXN3, NFASC, NLGN2*) was added to each well, except in the blank well. A sealing plate membrane was added and they were kept reacting for 60 min in a 37°C incubator. The liquid was dried in the hole and then washed five times with a washing solution. Then, it was patted dry with absorbent paper. Per well was added with 100 µl ABC working solution (50 μl of liquid A and 50 μl of liquid B). They were mixed gently and remained for 15 min in a 37°C incubator in darkness. Each well was added with a 50 μl stop solution, then the color changed from blue to yellow. The blank hole was used as a Zero set. The absorbance value (OD value) at 450 nm of each hole is measured with the enzyme plate analyzer, within 15 min of the completion of the previous step. The curve data fitting software “Curve Expert 1.3” was employed to plot the best‐fitting curve by the average absorbance and concentration of each standard (five times dilution). Through this standard curve, each sample concentration (µg/ml) was calculated. In general, the correlation coefficient of the standard ELISA curve reached above 0.99.

### 2.6 Statistical analysis

All data were statistically analyzed by the SPSS 20.0 software. When the continuous variable data conformed to the normal distribution, the mean ± standard deviation was used to carry out the *T* test of two independent samples. If it was non‐normal distribution, the median and interquartile range was employed to represent it. Wilcoxon's rank‐sum test was performed to compare two independent samples. The u^2^ test was used on the classification variables. Pearson's correlation analysis was employed for correlation analysis. It was considered statistically significant when *p* < 0.05.

## 3 RESULTS

### 3.1 The basic information of VE and control groups

The proteomics detection was performed in the CSF of 11 VE patients and 5 patients with non‐nervous system infection. The VE group included six males and five females, with an average age of 52.2 ± 3.05 years. The control group included two males and three females, with an average age of 42.63 ± 5.26 years. There was no statistically significant difference in age and gender between the two groups (*p* > 0.05).

### 3.2 Total protein function analysis

**Figure 2:**
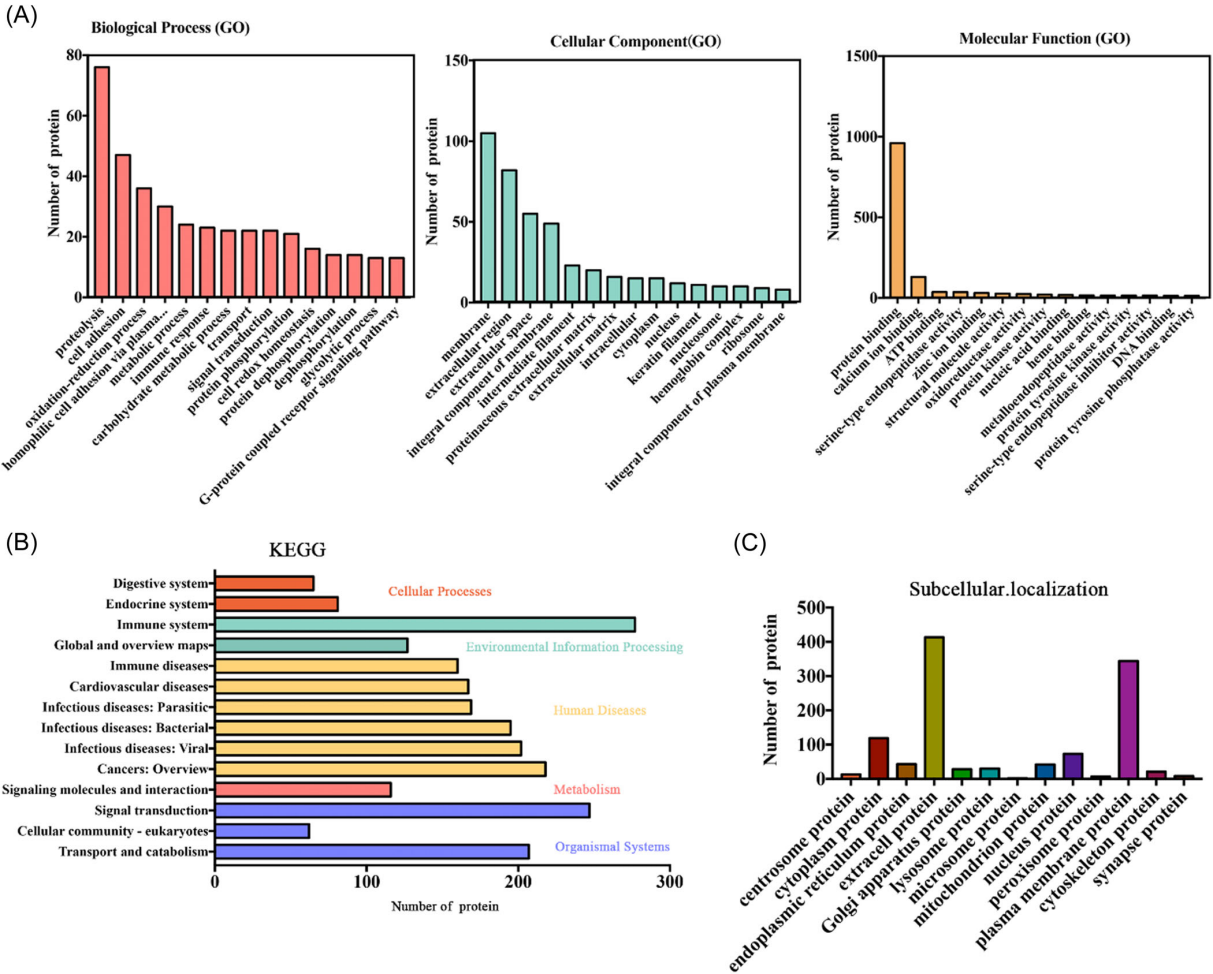
Overall functional analysis. All proteins identified in the sample were annotated in a common function database. (A) GO analysis. (B) KEGG analysis. (C) Subcellular localization information. GO, Gene Ontology; KEGG, Kyoto Encyclopedia of Genes and Genomes analysis. [Color figure can be viewed at wileyonlinelibrary.com]

Sixteen qualified CSF samples were screened for protein detection, including 11 in the VE group and 5 in the control group (Figure 2). All the proteins were analyzed, including GO analysis (Figure 2A), KEGG analysis (Figure 2B), and subcellular localization information (Figure 2C).

These proteins were grouped on the basis of GO domain annotations, including BP, MF, and CC (Figure 2A). The top five BPs were proteolysis, cell adhesion, oxidation–reduction process, homophilic cell adhesion via plasma membrane adhesion molecules, and metabolic process. The top five CCs were membrane, extracellular region, extracellular space, integral component of membrane, and intermediate filament. The top five MFs were protein binding, calcium ion binding, ATP binding, serine‐type endopeptidase activity, and zinc ion binding.

In the KEGG pathway, cellular processes included the endocrine system and the digestive system. Environmental information processing included immune system, and global and overview maps. Human diseases included cancers: overview; infectious diseases including bacterial, parasitic, and viral, cardiovascular diseases; and immune diseases. Metabolism included signaling molecules and interaction. Organismal systems included signal transduction, transport and catabolism, and cellular community—eukaryotes (Figure 2B).

The top five subcellular localization information of DEPs was mainly expressed in extracellular protein, plasma membrane protein, cytoplasmic protein, nucleus protein, and lysosome protein (Figure 2C).

### 3.3 Screening of DEPs

**Figure 3:**
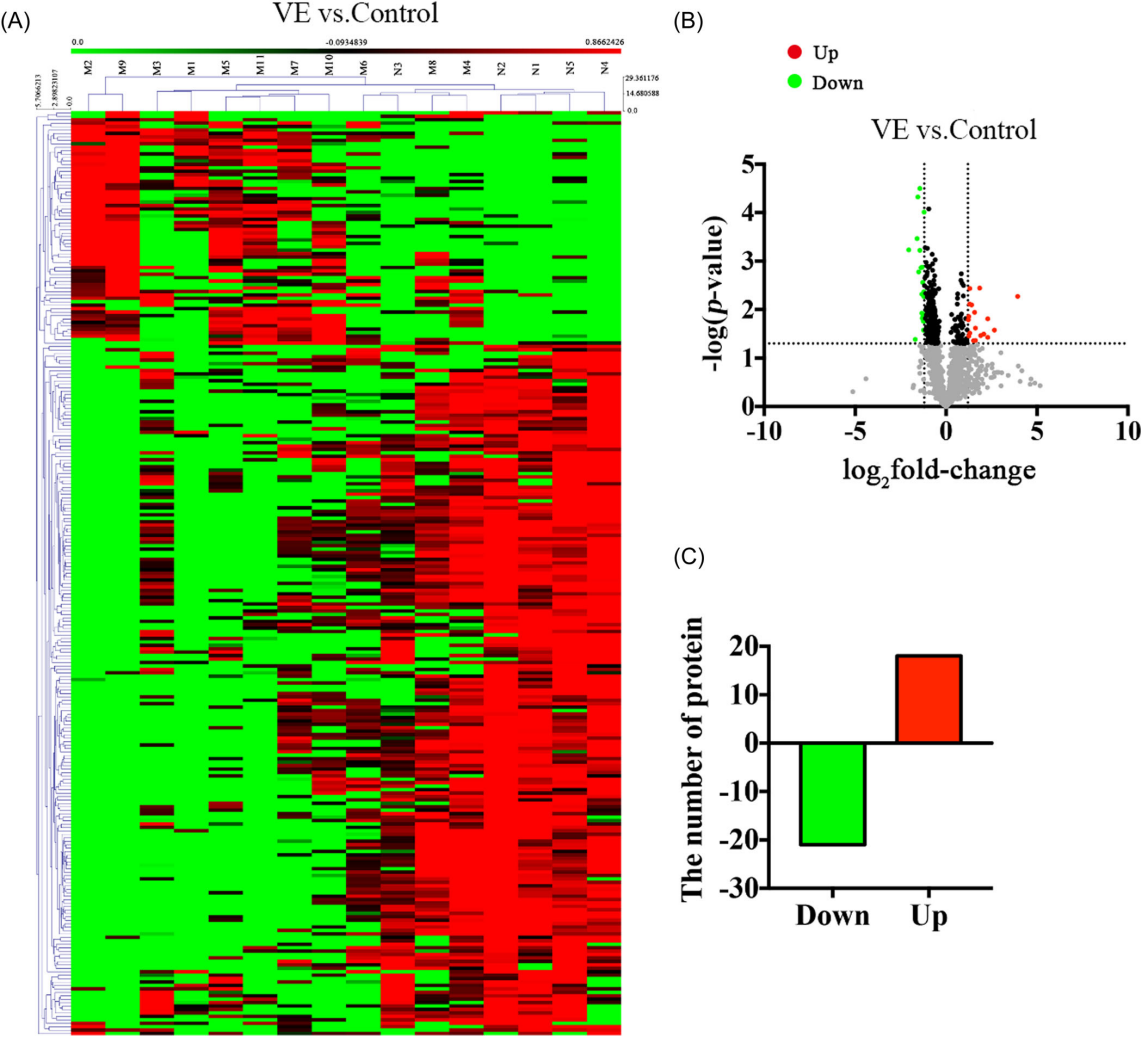
Differential proteins between VE and control groups. (A) Cluster analysis diagram of differential proteins between VE and control groups. (B) Volcano maps of DEPs with fold‐change greater than 1.2. (C) The number of specific fold‐change DEPs. DEP, differentially expressed protein; VE, viral encephalitis. [Color figure can be viewed at wileyonlinelibrary.com]

All proteins in the VE group and the control group were quantitatively analyzed, including the total difference identified proteins analysis, the screening and the clustering analysis of the different proteins, and the expression pattern clustering analysis. Cluster analysis was performed on the relative protein content of each sample, which was used to observe the upregulation and downregulation of different proteins when comparing between different samples (Figure 3A). A total of 39 DEPs were identified between two groups, including 18 upregulated proteins and 21 downregulated proteins (Figure 3B,C).

### 3.4 Functional analysis of DEPs

**Figure 4:**
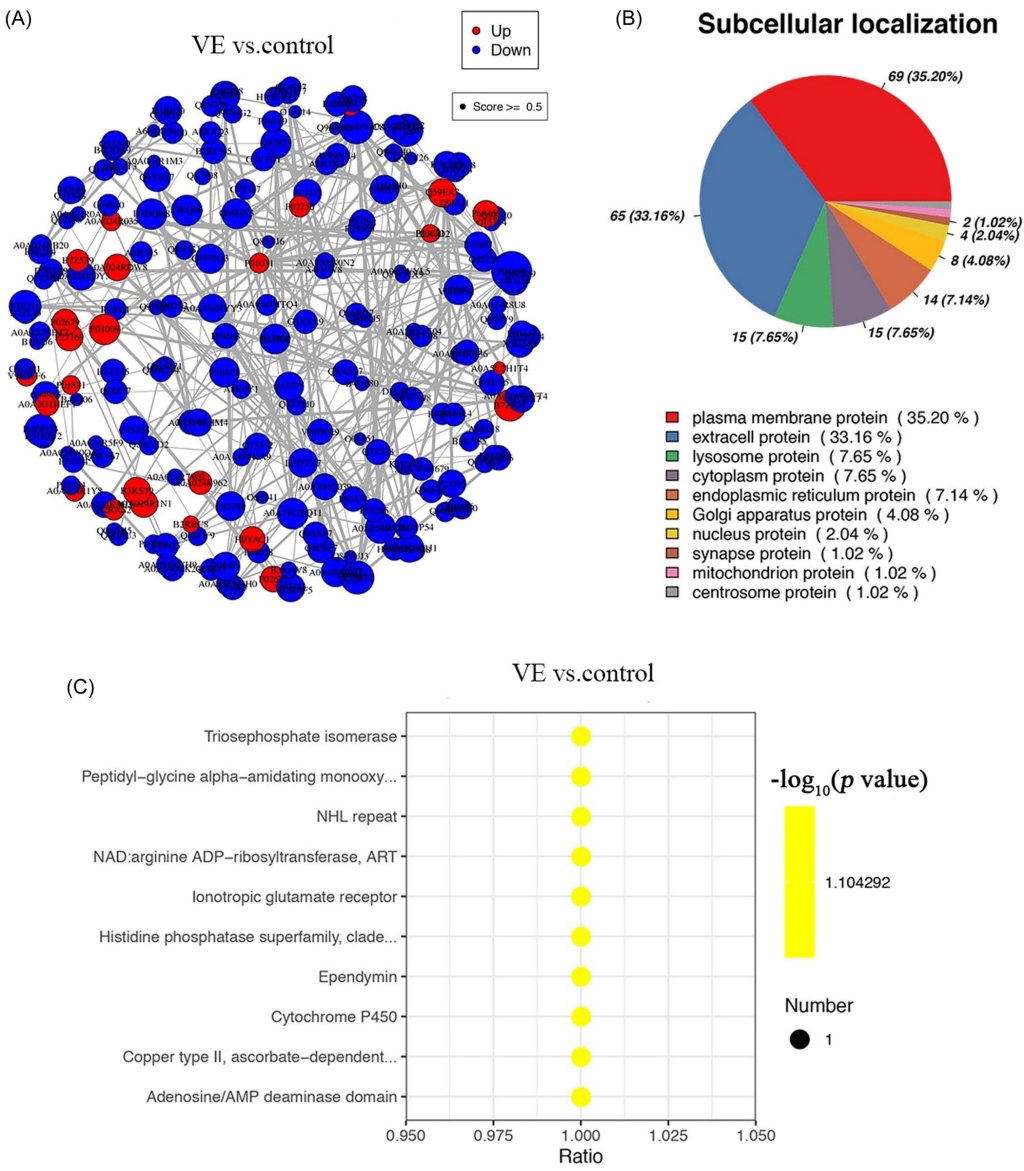
Functional analysis of DEPs. (A) The results of interaction analysis of PPI analysis of different proteins between VE and control groups. (B) Subcellular localization information of different proteins in VE and control groups. (C) The main domains of different proteins in VE and control groups. DEP, differentially expressed proteins; PPI, psychophysiological interaction; VE, viral encephalitis. [Color figure can be viewed at wileyonlinelibrary.com]

We plotted the psychophysiological interaction (PPI) analysis results of the DEPs of patients in the VE and control groups, where downregulated proteins were in the majority (Figure 4A).

According to subcellular localization, the DEPs were mainly plasma membrane protein (35.20%), extracellular protein (33.16%), lysosome protein (7.65%), cytoplasmic protein (7.65%), and endoplasmic reticulum protein (7.14%) (Figure 4B).

In addition, all DEPs were enriched with domain (IPR) analysis and the results showed that the DEPs were enriched in the triosephosphate isomerase, peptidyl‐glycine α‐amidating monooxy, NHL repeat, NAD: arginine ADP‐ribosyltransferase, ART, ionotropic glutamate receptor, histidine phosphatase superfamily, clade, ependymin, cytochrome P450, copper Type II, ascorbate‐dependent, adenosine/AMP deaminase domain, and so on (Figure 4C).

### 3.5 Screening and functional analysis of DEPs

**Figure 5:**
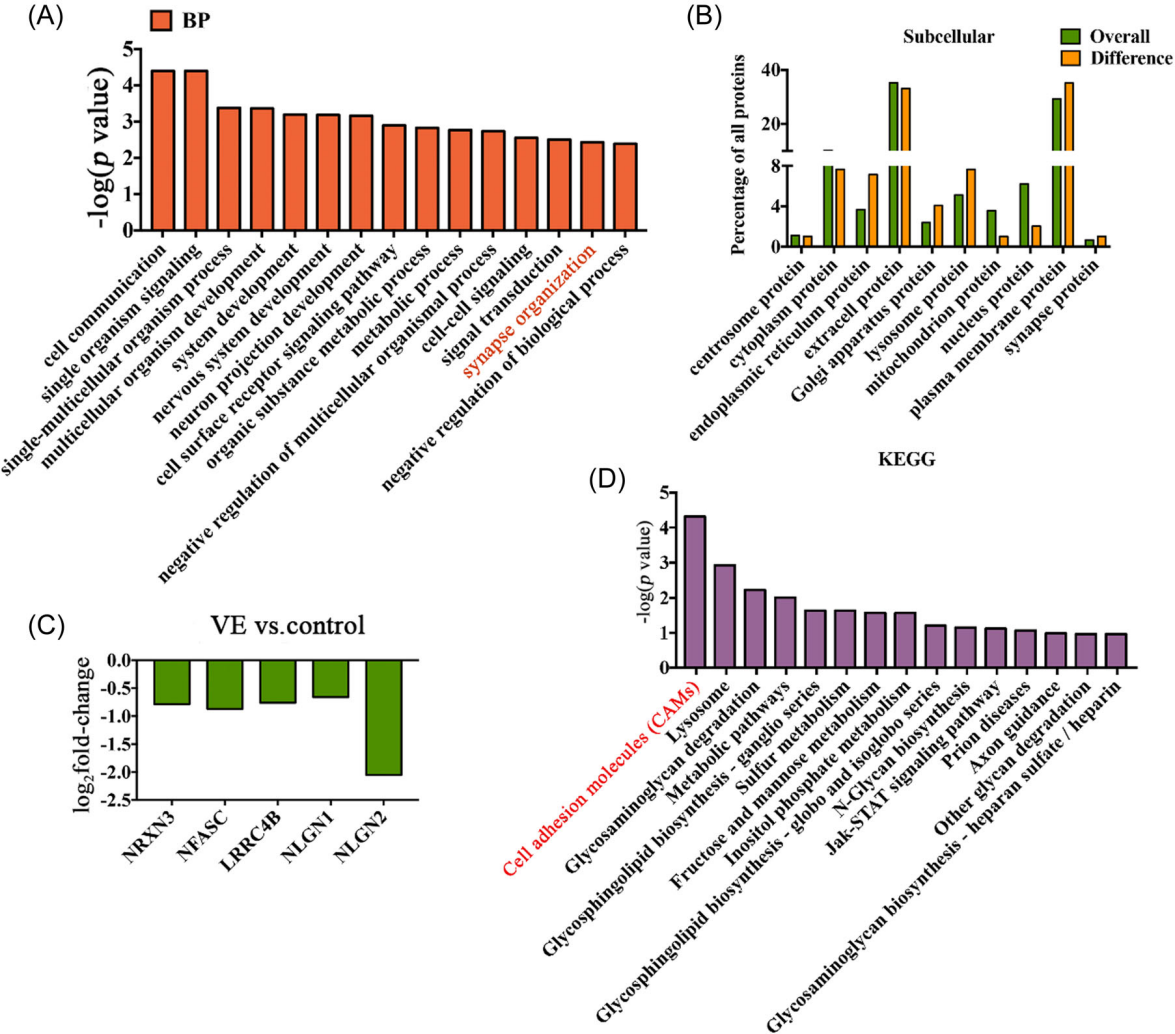
BP and KEGG analysis of DEPs. (A) GO analysis results of downregulated DEPS in the VE and control groups. (B) Comparison of protein nuclei and DEP subcellular localization. (C) Log_2_ fold‐change expression of proteins associated with the synapse organization. (D) KEGG analysis results of downregulated DEPs between VE and control groups. BP, biological process; DEP, differentially expressed proteins; GO, Gene Ontology; KEGG, Kyoto Encyclopedia of Genes and Genomes; VE, viral encephalitis. [Color figure can be viewed at wileyonlinelibrary.com]

The BP of GO analysis was performed for DEPs in the VE group and control group (Figure 5A). The top 15 BPs were cell communication, single organism signaling, single–multicellular organism process, multicellular organism development, system development, nervous system development, neuron projection development, cell surface receptor signaling pathway, organic substance metabolic process, metabolic process, negative regulation of the multicellular organismal process, cell–cell signaling, signal transduction, synapse organization, and negative regulation of BP.

By comparison of the subcellular location of DEPs detected, what accounted for an increased proportion were endoplasmic reticulum protein, Golgi apparatus protein, lysosome protein, plasma membrane protein, and synapse protein (Figure 5B).

The BP analysis and subcellular localization analysis showed that the proteins associated with axon tissue were significantly downregulated. Furthermore, log_2_ fold‐change expression analysis was performed on the proteins associated with synapse organization (axon tissue). The five proteins with the largest difference were selected, among which *NLGN2, NFASC, NRXN3, LRRC4B*, and *NLGN2* were significantly downregulated, and *NRXN3, NFASC*, and *NLGN2*  downregulated most significantly (Figure 5C).

KEGG enrichment analysis was performed for downregulated DEPs (Figure 5D). Histograms made for the top 15 pathways in the enrichment list showed that the enrichment pathway were cell adhesion molecules (CAMs), lysosome, glycosaminoglycan degradation, metabolic pathways, glycosphingolipid biosynthesis‐ganglio series, sulfur metabolism, fructose and mannose metabolism, inositol phosphate metabolism, glycosphingolipid biosynthesis‐globo and isoglobo series, N‐glycan biosynthesis, Jak‐STAT signaling pathway, prion diseases, axon guidance, other glycan degradation, and glycosaminoglycan biosynthesis‐heparan sulfate/heparin.

### 3.6 Basic data analysis of enlarged sample size in VE and control groups

CSF was collected from a total of 150 patients. According to the inclusion and exclusion criteria of each group, finally, 73 patients were included in the VE group and 53 cases in the control group.

There was no statistical significance in CSF pressure, CSF Cl, CSF Glu and LY (*p* = 0.543, *p* = .484, *p* = 0.701, *p* = 0.868, respectively). The statistically significant data exist in CSF WBC, CSF Pro, WBC, NEUT, and length of hospital stay (*p* = 0.01, *p* < 0.001, *p* = 0.001, *p* = 0.001, *p* < 0.001, respectively) (Table 2).

**Table 2:** Baseline data analysis.

	Total	VE group	Control group	*p* value
Patient	126	73	53	
Gender	126	Male (44)	Male (4)	0.096
		Female (29)	Female (29)	
Age	45 (29–61)	46.5 (28.75–65.25)	45 (31.5–56.5)	0.405
History of cold exposure	114 [90]	Yes: 27 [38]	Yes: 24 [46]	0.042*
		None: 45 [62]	None: 18 [34]	
Main symptoms	115	Headache: 31 [43]	Headache: 27 [51]	0.23
		Mental disorders: 25 [34]	Mental disorders: 8 [15]	
		Limb fatigue: 9 [12]	Limb fatigue: 4 [8]	
		Convulsions: 1 [1]	Convulsions: 0 [0]	
		Consciousness disorders: 7 [10]	Consciousness disorders: 3 [6]	
CSF brain pressure	145 (120–180)	147.5 (117.5–190)	145 (120–170)	0.543
Electroencephalogram	83 [66]	Normal: 25 [34]	Normal: 21 [40]	0.156
		Mild abnormality: 25 [34]	Mild abnormality: 7 [13]	
		Boundary: 2 [3]	Boundary: 1 [2]	
		Moderately abnormal: 1 [1]	Moderately abnormal: 0 [0]	
CSF WBC (/L)	1 (1–4)	2 (1–5.5)	1 (0–3)	0.01*
CSF Cl (mmol/L)	127.31 ± 3.05	127.14 ± 3.33	127.56 ± 2.50	0.484
CSF Glu (mmol/L)	3.58 (3.26–3.95)	3.62 (3.26–4.10)	3.51 (3.29–3.87)	0.701
CSF Pro (mg/L)	410 (303–550)	517 (404.5–641.75)	300 (242.5–348.5)	<0.001*
WBC (10^9^/L)	7.18 (5.57–8.81)	7.41 (6.20–9.28)	6.43 (4.83–7.84)	0.001*
LY (10^9^/L)	1.68 (1.17–2.21)	1.67 (1.12–2.22)	1.60 (1.19–2.17)	0.868
NEUT (10^9^/L)	4.35 (3.44–5.82）	4.68 (3.70–6.47)	3.71 (2.75–5.20)	0.001*
Days of hospitalization	11 (8–14)	12 (10–15)	8 (5.5–10)	<0.001*

*Note*: Compared with the control group.

Abbreviations: CSF Cl, cerebrospinal fluid chloride; CSF Cl, CSF chloride; CSF Glu, CSF glucose; CSF Pro, CSF protein; CSF WBC, CSF leukocyte; LY, lymphocyte absolute value; NEUT, neutrophils absolute value.

* Compared with the control group, *p* < 0.05

### 3.7 The proteins level of *NRXN3, NFASC*, and *NLGN2* in VE and control groups

**Figure 6:**
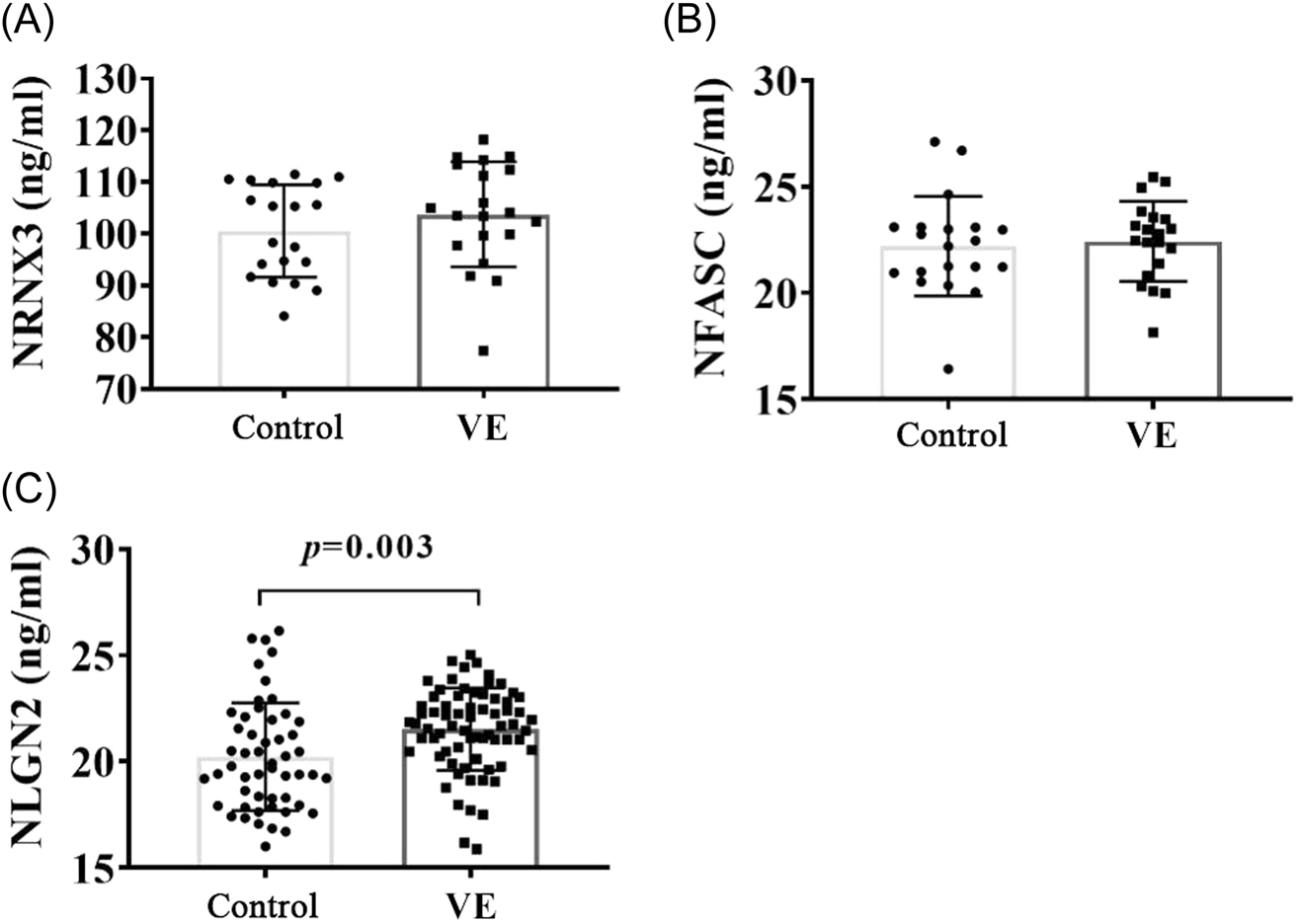
The expression of *NRXN3, NFASC*, and *NLGN2* was verified by ELISA in VE and control groups. (A) Comparison of *NRNX3* protein between VE and control groups (20 samples in each group).(B) Comparison of *NFASC* protein between VE and control groups (20 samples in each group). (C) Comparison of *NLGN2* protein between VE and control groups (with an increase in sample volume, 73 cases in the VE group, and 53 cases in the control group). ELISA, enzyme‐linked immunosorbent assay; *NFASC*, neurofascin; *NLGN2*, neuroligin 2; *NRXN3*, neurexin 3; VE, viral encephalitis.

*NRXN3, NFASC*, and *NLGN2* proteins were verified by ELISA in 20 samples of each group. There was no significant difference in the expression of *NRXN3* and *NFASC* in CSF between the two groups (*p* = 0.294, *p* = 0.744). The expression of *NLGN2* protein in the VE group was significantly higher than that in the control group, and then the sample volume was expanded to validate *NLGN2* protein. The expression of NLGN2 protein in the VE group was higher than that in the control group, which was statistically significant (*p* = 0.003) (Figure 6).

### 3.8 Correlation analysis of *NLGN2*

**Figure 7:**
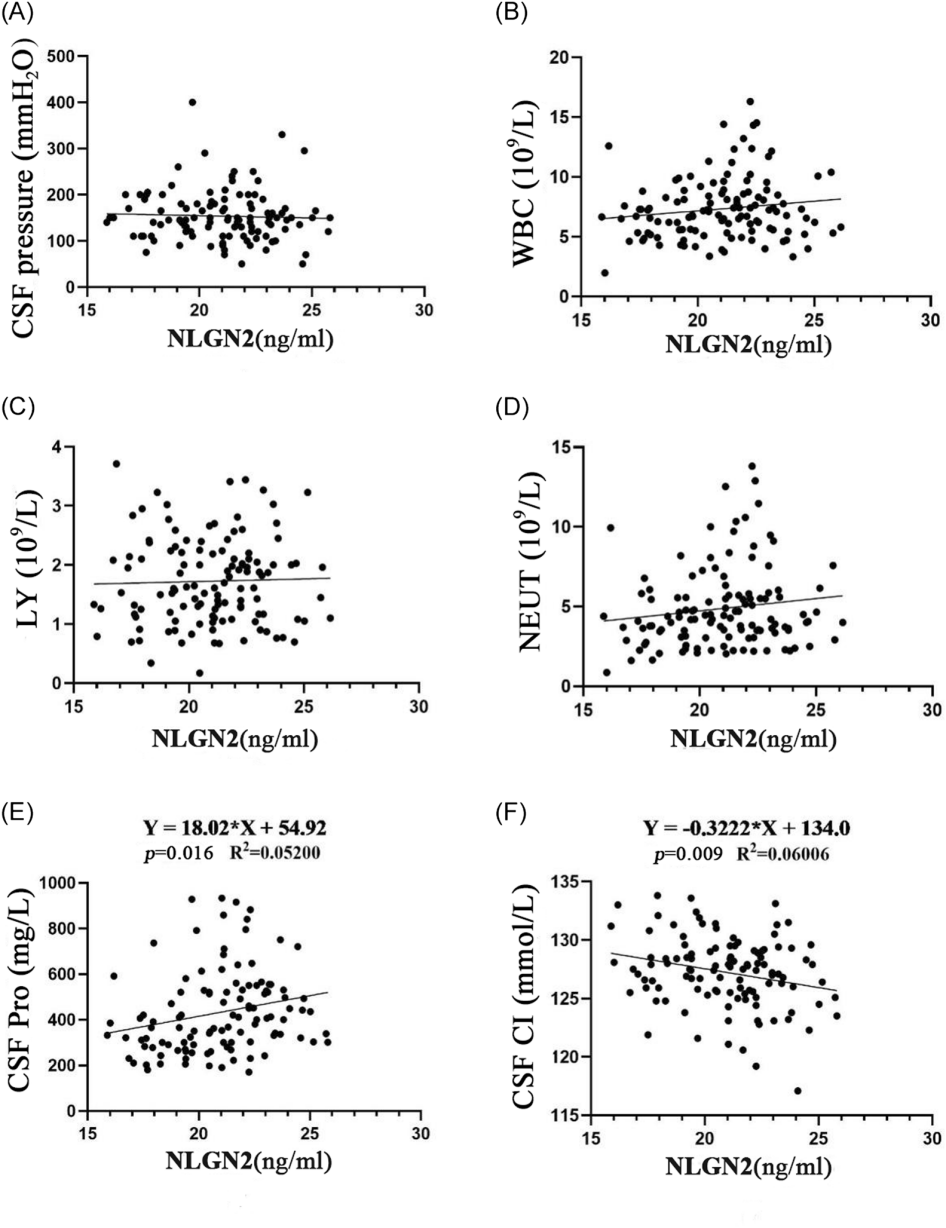
Correlation analysis of *NLGN2*. (A) Correlation analysis diagram of *NLGN2* protein and CSF pressure. (B) Correlation analysis diagram of *NLGN2* protein and WBC. (C) Correlation analysis diagram of *NLGN2* protein and LY. (D) Correlation analysis diagram of *NLGN2* protein and NEUT. (E) Correlation analysis diagram of *NLGN2* protein and CSF Pro. (F) Correlation analysis diagram of *NLGN2* protein and CSF Cl. CSF Cl, CSF chloride; CSF Pro, cerebrospinal fluid protein; LY, lymphocyte absolute value; *NLGN2*, neuroligin 2; NEUT, neutrophils absolute value; WBC, white blood cell.

It was found that *NLGN2* protein in CSF had no correlation with CSF pressure, WBC, LY, and NEUT (*p* = 0.666, *p* = 0.12, *p* = 0.75, *p* = 0.12), but was somewhat correlated with CSF Pro and CSF Cl (*p* = 0.016, *p* = 0.009) (Figure 7).

## 4 DISCUSSION

The atypical clinical manifestations of VE and the complexity of the pathogenesis lead to great limitations in the diagnosis. Because CSF is close to the CNS microenvironment and has relative protein content, it is preferred as an ideal CNS biomarker.^25^ In this study, label‐free proteomic analysis technology was performed to construct CSF differential proteomics between VE and control patients. ELISA was further employed to verify three downregulated proteins and analyze the clinical correlation. This study demonstrated that the identified DEPs proportion of the VE group and control group in CSF samples is consistent in BP, MF, and CC, indicating the relatively stable overall composition of CSF Pro.^23^ Based on the analysis of CSF Pro differential expression in VE patients by label‐free proteomics, we can improve our understanding of VE, find potential biomarkers, and provide a valuable reference for clinical diagnosis and treatment.

We analyzed the total protein in the CSF of the VE group and control group. The composition of BP involved in the main BPs includes proteolysis (23%), cell adhesion (14%), and oxidation–reduction process (11%). The composition of CC was mainly located in the plasma membrane (31%), extracellular region (24%), extracellular space (17%), and integral component of the membrane (15%). The composition of MF mainly includes protein binding (74%) and calcium ion binding (10%). KEGG pathway annotation is mainly involved in the immune system, signal transduction, transport and molecular metabolism, viral infectious diseases, and other signal transduction pathways in the occurrence of diseases. All proteins in VE and control groups were quantitatively analyzed by label‐free proteomics analysis, and then 39 DEPs were identified between the two groups. According to GO analysis on BP, CC, and MF processes, DEPs were mainly in connection with biological metabolic process, catalytic activity, cell process, and axon tissue, which may be related to inflammatory damage in nerve cells and axons. Next, according to KEGG analysis, the DEP enrichment pathway is mainly in CAMs, which participate in various pathophysiological processes, such as inflammatory reaction, cell signal transduction, immune response, and so on. Thereby it may play a significant role in VE pathogenesis. Then, through GO, subcellular localization, and KEGG enrichment analyses, it can be seen that the proteins related to axon tissue are obviously downregulated. By the difference analysis of axon‐related proteins, five proteins with the greatest differences were selected, among which *NRXN3, NFASC*, and *NLGN2* proteins may be closely related to VE pathogenesis.

Neuron cells contact each other through synapses and each synapse consists of a presynaptic membrane, postsynaptic membrane, and synaptic gap. The electrical activity of presynaptic neurons triggers neurotransmitters to be released into synaptic spaces, activating neuronal cells on postsynaptic membranes to undergo chemical reactions. Synapses, as transmissions of information in the brain, play a key role in establishing and maintaining neural pathways. There are amounts of synaptic adhesion/tissue molecules in the mammalian brain to provide information on recognition, transmission, and signal processing, playing a crucial part in cell adhesion and tissue protein interaction network. Through numerous proteomic studies and literature reviews, it is estimated that there are more than 1900–2700 proteins^28^, ^29^, ^30^ in synapses. These synaptic proteins exist in synaptic vesicle exocytosis and circulation, various neurotransmitter receptors, ion channels, cell matrix, CAMs, cytoskeleton, scaffold, membrane transporters, phosphatases, and proteins involved in protein degradation. Studies have demonstrated that hundreds of genes encoding synaptic proteins are associated with schizophrenia, autism spectrum disorders, and other cognitive disorders.^31^, ^32^, ^33^, ^34^

It was proved axonal injury is the main pathophysiological change of various CNS, such as amyotrophic lateral sclerosis, multiple sclerosis, Parkinson's disease, and peripheral neuropathy. Studies have testified that inflammation participates in the process of axonal injury, coping with the inflammatory response of inflammatory factors, tumor necrosis factor, and Toll‐like receptors by producing oxidative active substances.^35^, ^36^, ^37^ These active substances combine with oxides produced by cell damage and lipid peroxidation, thus resulting in changes in nuclear factor‐κb‐dependent transcriptional pathways and aggravating inflammatory damage.^38^ In the autoimmune encephalomyelitis mouse model, it is proved that neuroinflammation may lead to neurological dysfunction of MS and other neuroinflammatory diseases.^39^ In addition, macrophages also participate in the myelin sheath damage process, directly degrading myelin sheath protein by releasing protease.^40^ Therefore, the virus may participate in the inflammatory injury process by mediating the abnormal expression of axon‐related proteins. *NRXN3, NFASC*, and *NLGN2* proteins are axon‐related proteins. When expressed abnormally, they involve in axon injury. The details are as follows:

Neurexins (*NRXN*) is adhesion molecule proteins family in neuronal presynaptic cell. They combine with glial cells on the postsynaptic membrane and play a vital role in regulating the release of neuronal neurotransmitters at neuronal synapses.^41^ This protein family is mainly involved in cell recognition and cell adhesion, cell signal transduction, neurotransmitter release, and synapse formation. A large number of studies have confirmed that *NRXN* plays an important role in maintaining synaptic function in neuropsychiatric diseases and neurodevelopmental disorders.^42^, ^43^ Studies have revealed that knockout of the mouse‐*NRXN* gene leads to abnormal protein expression, impairs the presynaptic release of excitatory and inhibitory synapses, and leads to the reduction of cellular calcium current.^44^ After the knockout of the mouse *NRXN3* gene, presynaptic release at the olfactory bulb inhibitory synapse was significantly reduced.^41^ In addition, abnormal expression of *NRXN3* is related to autism, addiction, schizophrenia, Alzheimer's disease, and other diseases. Mutations in the *NRXN* gene lead to axonal growth and development disorders in autism spectrum diseases.^45^ A small number of studies have also suggested that *NRXN3* rearrangement may be related to cluster headaches in some cases.^46^ Subsequent studies also reported that abnormal expression and imbalance of presynaptic *NRXN3* may increase neuronal inflammation in the brain for AD patients.^47^

*NFASC* is a member of the immunoglobulin cell of the adhesion molecule L1 family and a transmembrane protein encoded by the *NFASC* gene.^48^ It is abundant in the adult CNS, especially in the cerebellum and peripheral nerves.^49^, ^50^ *NFASC* plays a crucial role in the development and function of the initial axonal segment and Ranvier node.^51^ Meanwhile, it plays an important role in maintaining the cytoskeleton of glial cells and neurons.^52^ Nerve conduction in vertebrates relies on rapid impulse transmission by high concentration voltage‐gated sodium channels accumulated between the Ranvier node of myelinated nerve fibers. Therefore, *NFASC* plays a vital role in the development and function of central and peripheral nervous systems, and its dysfunction may lead to nervous system diseases. Abnormal expression of *NFASC* leads to neurodevelopmental abnormalities, including neurodevelopmental disorders and demyelinating neuropathy in the central and peripheral. These diseases are caused by abnormal expression of cell adhesion protein, involving nerve sheath extension, axon guidance, synapsis, myelin sheath formation, and neuron–glial cell interaction. Studies have reported that abnormal expression of *NFASC* is associated with chronic inflammatory demyelinating polyneuropathy and causes immune central accessory sarcoidosis.^53^

Neuroligin (*NLGN*) is a postsynaptic cell adhesion protein molecule that interacts with *NRXN* to participate in synaptic formation and function.^43^ *NLGN2* exists throughout the CNS and is mainly closely related to inhibitory GABA aminobutyric acid signals, which are crucial to maintaining the balance of excitation and inhibition in the brain.^54^ Recent studies have confirmed that *NLGN2* is a component of inhibitory synapses in medium‐sized multispinous neurons and is expressed in GABA‐ergic neurons.^55^, ^56^ The imbalance of *NLGN2* excitatory and inhibitory neuronal signals causes a series of neuropsychiatric diseases. Studies on *NLGN2* gene knockout mice showed that *NLGN2* has unique functions in maintaining inhibitory synaptic function and regulating anxiety behavior.^57^, ^58^, ^59^ Abnormal expression of *NLGN2* will lead to anxiety, growth retardation, dyskinesia, social disorders, aggressive and sensory processing defects, and changes in social skills in animal models.^60^ Analysis of *NLGN* transcription genes in patients with death from severe depressive disorder suggested that the expression of *NLGN2* was downregulated.^61^

Based on label‐free proteomics, this study further verified *NRXN3, NFASC*, and *NLGN2* proteins by ELISA. Univariate analysis of VE and control groups indicated that CSF pressure, CSF Pro, absolute value of blood leukocytes, absolute value of blood neutrophils, and hospitalization days were related to the severity of the disease. Analysis of *NRXN3, NFASC*, and *NLGN2* proteins in CSF of the two groups revealed that the expression of *NLGN2* protein in the VE group was prominent, which indicated that *NLGN2* participates in the pathogenesis of VE. Studies have shown that *NLGN2* participates in synapse formation and function. Inflammation may lead to abnormal protein expression by damaging axons in the process of virus infection. Hence, there are reasons why *NLGN2* content decreased in CSF of VE patients in this experiment. Pathogens directly damage axon tissue and participate in the process of virus infection, resulting in the destruction of *NLGN2* protein. Moreover, it is also possible to participate in the immune response by directly binding to the virus. From the above process, *NLGN2* is consumed in large quantities, resulting in a decrease in content. However, the reason for the decrease of *NLGN2* protein in CSF of VE remains unclear, and the specific mechanism requires further study. The verification results of *NRXN3* and *NFASC* protein content are opposite to proteomic analysis results. Proteomic results suggest downregulation, but upregulation in ELISA verification results. This may be due to the immune response caused by a virus infection, and consequently, the body produces a corresponding protective response to slow down inflammatory damage. The correlation analysis of *NLGN2* protein in the VE group suggested that the patient's condition may be relatively mild, causing CSF changes slightly, with protein and other changes expressed scarcely. This study provides a reference direction for further elucidation of the VE pathogenesis, and provides some new candidate molecules for early diagnosis of VE, improving the accuracy of diagnosis and prognosis. Nonetheless, in view of the possible limitations in the design, technology, and analysis strategy of this study, we need to further expand the sample size, multigroup science, and adopt various verification methods, such as PCR and Western blot, to confirm the reliability of these research results.

## 5 CONCLUSIONS

Differential proteins in cerebrospinal fluid of VE patients were screened by label‐free proteomics and bioinformatics analysis was carried out. Further ELISA experiment verified that *NLGN2* protein was differentially expressed in VE and control groups, and positively correlated with CSF Pro. Based on the validation results, *NLGN2* is expected to be a candidate biomarker for the early diagnosis of VE.

## AUTHOR CONTRIBUTIONS

Qian Wang contributed the central idea. Qian Wang, Shan‐Shan Yan, and Jun‐Yan Zhang conceived and designed the experiments. Qian Wang, Ruo‐Lan Du, and Lu‐Lu Xue analyzed most of the data. Qian Wang, Shan‐Shan Yan, and Jun‐Yan Zhang wrote the initial draft of the paper. Chang‐Yin Yu and Juan Li contributed to carrying out additional analyses, refining the ideas, and finalizing this paper.

## CONFLICTS OF INTEREST

The authors declare no conflicts of interest.

## ETHICS STATEMENT

In the present study, experimental procedures were reviewed following the standard biosecurity and institutional safety procedures. The project was approved by the Ethics Committees of Kunming Medical University in Kunming, China (ethics no. KLLY‐2020‐001). All experiments were conducted in accordance with the World Medical Association Declaration of Helsinki. Written consent was signed for all patients aged 18 years or above. For patients under the age of 18 years, a legal guardian or parent signed the consent form.

## Data Availability

Research data are not shared.

## References

[1] Im JH , Baek J , Durey A , Kwon HY , Chung MH , Lee JS . Current status of tick‐borne diseases in South Korea. Vector‐Borne and Zoonotic Dis. 2019;19(4):225‐233. 10.1089/vbz.2018.2298 30328790

[2] Kadambari S , Harvala H , Simmonds P , Pollard AJ , Sadarangani M . Strategies to improve detection and management of human parechovirus infection in young infants. Lancet Infect Dis. 2019;19(2):e51‐e58. 10.1016/s1473-3099(18)30288-3 30322791

[3] Blom K , Cuapio A , Sandberg JT , et al. Cell‐mediated immune responses and immunopathogenesis of human tick‐borne encephalitis virus‐infection. Front Immunol. 2018;9:2174. 10.3389/fimmu.2018.02174 30319632 PMC6168641

[4] Hinson VK , Tyor WR . Update on viral encephalitis. Curr Opin Neurol. 2001;14(3):369‐374. 10.1097/00019052-200106000-00017 11371762

[5] Kumar B , Manuja A , Gulati BR , Virmani N , Tripathi BN . Zoonotic viral diseases of equines and their impact on human and animal health. Open Virol J. 2018;12:80‐98. 10.2174/1874357901812010080 30288197 PMC6142672

[6] Silva ASG , Matos ACD , Cunha MACR , et al. West Nile virus associated with equivalent encephalitis in Brazil, 2018. Transbound Emerg Dis. 2019;66(1):445‐453. 30318735 10.1111/tbed.13043

[7] Soung A , Klein RS . Viral encephalitis and neurologic diseases: focus on astrocytes. Trends Mol Med. 2018;24(11):950‐962. 10.1016/j.molmed.2018.09.001 30314877 PMC6546292

[8] Lakeman FD , Whitley RJ . Diagnosis of herpes simplex encephalitis: application of polymerase chain reaction to cerebrospinal fluid from brain‐biopsied patients and correlation with disease. National Institute of Allergy and Infectious Diseases Collaborative Antiviral Study Group. J Infect Dis. 1995;171(4):857‐863. 10.1093/infdis/171.4.857 7706811

[9] Stahl JP , Azouvi P , Bruneel F , et al. Guidelines on the management of infectious encephalitis in adults. Med Mal Infect. 2017;47(3):179‐194. 10.1016/j.medmal.2017.01.005 28412044

[10] Glaser CA , Honarmand S , Anderson LJ , et al. Beyond viruses: clinical profiles and etiologies associated with encephalitis. Clin Infect Dis. 2006;43(12):1565‐1577. 10.1086/509330 17109290

[11] Mailles A , Stahl JP . Infectious encephalitis in France in 2007: a national prospective study. Clin Infect Dis. 2009;49(12):1838‐1847. 10.1086/648419 19929384

[12] de Ory F , Avellón A , Echevarría JE , et al. Viral infections of the central nervous system in Spain: a prospective study. J Med Virol. 2013;85(3):554‐562. 10.1002/jmv.23470 23239485

[13] Kupila L , Vuorinen T , Vainionpää R , Hukkanen V , Marttila RJ , Kotilainen P . Etiology of aseptic meningitis and encephalitis in an adult population. Neurology. 2006;66(1):75‐80. 10.1212/01.wnl.0000191407.81333.00 16401850

[14] de Buhr N , Reuner F , Neumann A , et al. Neutrophil extracellular trap formation in the *Streptococcus suis*‐infected cerebrospinal fluid compartment. Cell Microbiol. 2017;19(2)​, 10.1111/cmi.12649 27450700

[15] Craft S , Peskind E , Schwartz MW , Schellenberg GD , Raskind M , Porte D Jr. Cerebrospinal fluid and plasma insulin levels in Alzheimer's disease: relationship to severity of dementia and apolipoprotein E genotype. Neurology. 1998;50(1):164‐168. 10.1212/wnl.50.1.164 9443474

[16] Fowler Å , Ygberg S , Bogdanovic G , Wickström R . Biomarkers in cerebrospinal fluid of children with tick‐borne encephalitis: association with long‐term outcome. Pediatr Infect Dis J. 2016;35(9):961‐966. 10.1097/inf.0000000000001210 27187756

[17] Asara JM , Christofk HR , Freimark LM , Cantley LC . A label‐free quantification method by MS/MS TIC compared to SILAC and spectral counting in a proteomics screen. Proteomics. 2008;8(5):994‐999. 10.1002/pmic.200700426 18324724

[18] Bantscheff M , Schirle M , Sweetman G , Rick J , Kuster B . Quantitative mass spectrometry in proteomics: a critical review. Anal Bioanal Chem. 2007;389(4):1017‐1031. 10.1007/s00216-007-1486-6 17668192

[19] Davidsson P , Paulson L , Hesse C , Blennow K , Nilsson CL . Proteome studies of human cerebrospinal fluid and brain tissue using a preparative two‐dimensional electrophoresis approach prior to mass spectrometry. Proteomics. 2001;1(3):444‐452. 10.1002/1615-9861(200103)1:3<444::Aid-prot444>3.0.Co;2-q 11680889

[20] Rohlff C . Proteomics in molecular medicine: applications in central nervous systems disorders. Electrophoresis. 2000;21(6):1227‐1234. 10.1002/(sici)1522-2683(20000401)21:6<1227::Aid-elps1227>3.0.Co;2-l 10786895

[21] Zheng PP , Luider TM , Pieters R , et al. Identification of tumor‐related proteins by proteomic analysis of cerebrospinal fluid from patients with primary brain tumors. J Neuropathol Exp Neurol. 2003;62(8):855‐862. 10.1093/jnen/62.8.855 14503641

[22] Gineste C , Ho L , Pompl P , Bianchi M , Pasinetti GM . High‐through protein and protein biomarker discovery in an empirical model of inflammable hypergesia: effects of nimesulide. Drugs. 2003;63(1):23‐29. 10.2165/00003495-200363001-00004 14506908

[23] Gong Y , Pu W , Jin H , et al. Quantitative proteomics of CSF reveals potential predicted biomarkers for extranodal NK‐/T‐cell lymphoma of nasal‐type with ethmoidal sinus metastasis. Life Sci. 2018;198:94‐98. 10.1016/j.lfs.2018.02.035 29496492

[24] Jones P , Binns D , Chang HY , et al. InterProScan 5: genome‐scale protein function classification. Bioinformatics. 2014;30(9):1236‐1240. 10.1093/bioinformatics/btu031 24451626 PMC3998142

[25] Collins MA , An J , Hood BL , Conrads TP , Bowser RP . Label‐free LC‐MS/MS proteomic analysis of cerebrospinal fluid identifies protein/pathway alterations and candidate biomarkers for amyotrophic lateral sclerosis. J Proteome Res. 2015;14(11):4486‐4501. 10.1021/acs.jproteome.5b00804 26401960 PMC5592736

[26] Huang da W , Sherman BT , Lempicki RA . Bioinformatics enrichment tools: paths toward the comprehensive functional analysis of large gene lists. Nucleic Acids Res. 2009;37(1):1‐13. 10.1093/nar/gkn923 19033363 PMC2615629

[27] Franceschini A , Szklarczyk D , Frankild S , et al. STRING v9.1: protein–protein interaction networks, with increased coverage and integration. Nucleic Acids Res. 2013;41(Database issue):D808‐D815. 10.1093/nar/gks1094 23203871 PMC3531103

[28] Croning MD , Marshall MC , McLaren P , Armstrong JD , Grant SG . G2Cdb: the Genes to Cognition database. Nucleic Acids Res. 2009;37(Database issue):D846‐D851. 10.1093/nar/gkn700 18984621 PMC2686544

[29] Pielot R , Smalla KH , Müller A , et al. SynProt: a database for proteins of detergent‐resistant synaptic protein preparations. Front Synaptic Neurosci. 2012;4:1. 10.3389/fnsyn.2012.00001 22737123 PMC3382120

[30] Pirooznia M , Wang T , Avramopoulos D , et al. SynaptomeDB: an ontology‐based knowledgebase for synaptic genes. Bioinformatics. 2012;28(6):897‐899. 10.1093/bioinformatics/bts040 22285564 PMC3307115

[31] Bourgeron T . Current knowledge on the genetics of autism and propositions for future research. C R Biol. 2016;339(7‐8):300‐307. 10.1016/j.crvi.2016.05.004 27289453

[32] Fromer M , Pocklington AJ , Kavanagh DH , et al. De novo mutations in schizophrenia implicate synaptic networks. Nature. 2014;506(7487):179‐184. 10.1038/nature12929 24463507 PMC4237002

[33] Hall J , Trent S , Thomas KL , O'Donovan MC , Owen MJ . Genetic risk for schizophrenia: convergence on synaptic pathways involved in plasticity. Biol Psychiatry. 2015;77(1):52‐58. 10.1016/j.biopsych.2014.07.011 25152434

[34] Schizophrenia Working Group of the Psychiatric Genomics Consortium . Biological insights from 108 schizophrenia‐associated genetic loci. Nature. 2014;511(7510):421‐427. 10.1038/nature13595 25056061 PMC4112379

[35] Mittal M , Siddiqui MR , Tran K , Reddy SP , Malik AB . Reactive oxygen species in inflammation and tissue injury. Antioxid Redox Signal. 2014;20(7):1126‐1167. 10.1089/ars.2012.5149 23991888 PMC3929010

[36] Nathan C , Cunningham‐Bussel A . Beyond oxidative stress: an immunologist's guide to reactive oxygen species. Nat Rev Immunol. 2013;13(5):349‐361. 10.1038/nri3423 23618831 PMC4250048

[37] Ramalingam M , Kim SJ . Reactive oxygen/nitrogen species and their functional correlations in neurodegenerative diseases. J Neural Transmiss. 2012;119:891‐910. 918. 10.1007/s00702-011-0758-7 22212484

[38] Gloire G , Legrand‐Poels S , Piette J . NF‐kappaB activation by reactive oxygen species: fifteen years later. Biochem Pharmacol. 2006;72(11):1493‐1505. 10.1016/j.bcp.2006.04.011 16723122

[39] Sadeghian M , Mastrolia V , Rezaei Haddad A , et al. Mitochondrial dysfunction is an import cause of multiple sclerosis. Sci Rep. 2016;6:33249. 27624721 10.1038/srep33249 PMC5021937

[40] Norton WT , Cammer W , Bloom BR , Gordon S . Neutral proteins secured by macrophages grade basic protein: a possible mechanism of inflammatory demyelination. Adv Exp Med Biol. 1978;100:365‐381. 80946 10.1007/978-1-4684-2514-7_26

[41] Aoto J , Földy C , Ilcus SM , Tabuchi K , Südhof TC . Distinct circuit‐dependent functions of presynaptic neurexin‐3 at GABAergic and glutamatergic synapses. Nat Neurosci. 2015;18(7):997‐1007. 10.1038/nn.4037 26030848 PMC4482778

[42] Südhof TC . Neuroligins and neurexins link synaptic function to cognitive disease. Nature. 2008;455(7215):903‐911. 10.1038/nature07456 18923512 PMC2673233

[43] Reissner C , Runkel F , Missler M . Neurexins. Genome Biol. 2013;14(9):213. 10.1186/gb-2013-14-9-213 24083347 PMC4056431

[44] Zhang W , Rohlmann A , Sargsyan V , et al. Extracellular domains of alpha‐neurexins participate in regulating synaptic transmission by selectively affecting N‐ and P/Q‐type Ca^2+^ channels. J Neurosci. 2005;25(17):4330‐4342. 10.1523/jneurosci.0497-05.2005 15858059 PMC6725120

[45] Vaags AK , Lionel AC , Sato D , et al. Rare deletions at the neurexin 3 locus in autism spectrum disorder. Am J Hum Genet. 2012;90(1):133‐141. 10.1016/j.ajhg.2011.11.025 22209245 PMC3257896

[46] Zarrilli F , Tomaiuolo R , Ceglia C , et al. Molecular analysis of cluster headache. Clin J Pain. 2015;31(1):52‐57. 24469609 10.1097/AJP.0000000000000075

[47] Hishimoto A , Pletnikova O , Lang DL , Troncoso JC , Egan JM , Liu QR . Neurexin 3 transmembrane and soluble isoform expression and splicing haplotype are associated with neuron inflammasome and Alzheimer's disease. Alzheimer's Res Ther. 2019;11(1):28. 10.1186/s13195-019-0475-2 30902061 PMC6429815

[48] Ango F , di Cristo G , Higashiyama H , Bennett V , Wu P , Huang ZJ . Ankyrin‐based subcellular gradient of neurofascin, an immunoglobulin family protein, directs GABAergic innervation at Purkinje axon initial segment. Cell. 2004;119(2):257‐272. 10.1016/j.cell.2004.10.004 15479642

[49] Thul PJ , Lindskog C . The human protein atlas: a spatial map of the human proteome. Protein Sci. 2018;27(1):233‐244. 10.1002/pro.3307 28940711 PMC5734309

[50] Carithers LJ , Moore HM . The Genotype‐Tissue Expression (GTEx) Project. Biopreserv Biobank. 2015;13(5):307‐308. 10.1089/bio.2015.29031.hmm 26484569 PMC4692118

[51] Monfrini E , Straniero L , Bonato S , et al. Neurofascin (NFASC) gene mutation causes autosomal recessive ataxia with demyelinating neuropathy. Parkinsonism Relat Disord. 2019;63:66‐72. 10.1016/j.parkreldis.2019.02.045 30850329

[52] Leshchyns'ka I , Sytnyk V . Reciprocal interactions between cell adhesion molecules of the immunoglobulin superfamily and the cytoskeleton in neurons. Front Cell Dev Biol. 2016;4:9. 10.3389/fcell.2016.00009 26909348 PMC4754453

[53] Delmont E , Manso C , Querol L , et al. Autoantibodies to nodal isoforms of neurofascin in chronic inflammatory demyelinating polyneuropathy. Brain. 2017;140(7):1851‐1858. 10.1093/brain/awx124 28575198

[54] Bang ML , Owczarek S . A matter of balance: role of neurexin and neuroligin at the synapse. Neurochem Res. 2013;38(6):1174‐1189. 10.1007/s11064-013-1029-9 23559421

[55] Rothwell PE , Fuccillo MV , Maxeiner S , et al. Autism‐associated neuroligin‐3 mutations commonly impair striatal circuits to boost repetitive behaviors. Cell. 2014;158(1):198‐212. 10.1016/j.cell.2014.04.045 24995986 PMC4120877

[56] Uchigashima M , Ohtsuka T , Kobayashi K , Watanabe M . Dopamine synapse is a neuroligin‐2‐mediated contact between dopaminergic presynaptic and GABAergic postsynaptic structures. Proc Natl Acad Sci USA. 2016;113(15):4206‐4211. 10.1073/pnas.1514074113 27035941 PMC4839454

[57] Varoqueaux F , Aramuni G , Rawson RL , et al. Neuroligins determine synapse maturation and function. Neuron. 2006;51(6):741‐754. 10.1016/j.neuron.2006.09.003 16982420

[58] Gibson JR , Huber KM , Südhof TC . Neuroligin‐2 deletion selectively decreases inhibitory synaptic transmission originating from fast‐spiking but not from somatostatin‐positive interneurons. J Neurosci. 2009;29(44):13883‐13897. 10.1523/jneurosci.2457-09.2009 19889999 PMC2814361

[59] Babaev O , Botta P , Meyer E , et al. Neuroligin 2 deletion alters inhibitory synapse function and anxiety‐associated neuronal activation in the amygdala. Neuropharmacology. 2016;100:56‐65. 10.1016/j.neuropharm.2015.06.016 26142252

[60] Parente DJ , Garriga C , Baskin B , et al. Neuroligin 2 nonsense variant associated with anxiety, autism, intellectual disability, hyperphagia, and obesity. Am J Med Genet A. 2017;173(1):213‐216. 10.1002/ajmg.a.37977 27865048

[61] Heshmati M , Aleyasin H , Menard C , et al. Cell‐type‐specific role for nucleus accumbens neuroligin‐2 in depression and stress susceptibility. Proc Natl Acad Sci USA. 2018;115(5):1111‐1116. 10.1073/pnas.1719014115 29339486 PMC5798379

